# Genome-wide expression profiling and phenotypic evaluation of European maize inbreds at seedling stage in response to heat stress

**DOI:** 10.1186/s12864-015-1282-1

**Published:** 2015-02-25

**Authors:** Felix P Frey, Claude Urbany, Bruno Hüttel, Richard Reinhardt, Benjamin Stich

**Affiliations:** Max Planck Institute for Plant Breeding Research, Carl-von-Linné-Weg 10, Köln, 50829 Germany; Max Planck-Genome-Centre, Carl-von-Linné-Weg 10, Köln, 50829 Germany

**Keywords:** Climate change, Zea mays, Heat tolerance, Genetic variation, Transcriptome, Natural phenotypic diversity

## Abstract

**Background:**

Climate change will lead in the future to an occurrence of heat waves with a higher frequency and duration than observed today, which has the potential to cause severe damage to seedlings of temperate maize genotypes. In this study, we aimed to (I) assess phenotypic variation for heat tolerance of temperate European Flint and Dent maize inbred lines, (II) investigate the transcriptomic response of temperate maize to linearly increasing heat levels and, (III) identify genes associated with heat tolerance in a set of genotypes with contrasting heat tolerance behaviour.

**Results:**

Strong phenotypic differences with respect to heat tolerance were observed between the examined maize inbred lines on a multi-trait level. We identified 607 heat responsive genes as well as 39 heat tolerance genes.

**Conclusion:**

Our findings indicate that individual inbred lines developed different genetic mechanisms in response to heat stress. We applied a novel statistical approach enabling the integration of multiple genotypes and stress levels in the analysis of abiotic stress expression studies.

**Electronic supplementary material:**

The online version of this article (doi:10.1186/s12864-015-1282-1) contains supplementary material, which is available to authorized users.

## Background

Silage maize (*Zea mays L.*) is of increasing importance [1] as predominantly used biogas substrate in Germany [2]. Sowing in early summer after cereals leads to an exposure of the seedlings to high temperature and potentially heat stress [3]. Temperate maize genotypes are severely damaged when temperature rises over an optimum level [4] and yields of maize are heavily reduced at temperatures above 30°C, which was shown for US maize germplasm [5]. Besides the seedling stage, heat stress during flowering and corn filling as well has severe impacts on maize cultivation [6].

Climate predictions suggest that the mean global temperature and variance of the temperature are expected to increase in the future [7]. This will cause globally in the future an occurrence of heat waves with a higher frequency and duration than observed today [8]. This in turn leads in the future to an increase of the duration and intensity of heat stress situations in cropping systems.

In response to heat stress, plants show various symptoms, including scorching (burning) of leaves as well as growth inhibition and reduction of yield [9], which also has been reported for maize in temperate regions [4]. Improving maize genotypes to be able to cope with high temperatures leads to high reduction of yield losses due to climate change [10]. In this respect, the development of heat tolerant varieties is a major challenge for plant scientists and is of crucial importance for future maize cropping in temperate regions. The latter can be facilitated by gaining knowledge of the molecular basis of heat response and tolerance in maize. Furthermore, knowledge on the heat tolerance of European Flint and Dent lines is rare and highly valuable for plant breeding.

Recently, the understanding of the molecular response upon heat stress in plants in general and in maize in particular has increased (see reviews of [9,11] and [12]). The primarily major adverse effects of heat stress on plants are the decreased stability of membranes [13] and proteins, the excessive production of reactive oxygen species, a loss of cellular water, and an alteration of enzymatic reactions [12]. These changes lead especially to oxidative stress, impairment of metabolite synthesis, disturbed osmotic potential, and cell organization, to leaf burning, premature senescence, reduced growth, and cell death [12]. To cope with these adverse effects, plants developed several heat tolerance mechanisms (reviewed by [14]). They include the alteration of signaling cascades and transcriptional control, increasing production of antioxidants [15-17] and osmoprotectants, as well as the expression of stress proteins [12], especially heat shock proteins. We hypothesize that increasing heat stress is followed by a strong common transcriptomic response across different maize genotypes and that, however, certain genes exist, which are differentially regulated between genotypes with different heat tolerance.

Despite the high number of studies examining the molecular response of plants upon heat stress, most of the studies focused on the heat response of one or few genotypes and, thus, results are based on a narrow genetic background. Furthermore, to the best of our knowledge, all previous studies compared one standard condition with one heat level, but information about the behaviour of genotypes across a gradient of heat conditions is missing.

The objectives of this study were to (I) assess phenotypic variation for heat tolerance of temperate European Flint and Dent maize inbred lines, (II) investigate the transcriptomic response of temperate maize to linearly increasing heat levels and, (III) identify genes associated with heat tolerance in a set of genotypes with contrasting heat tolerance behaviour.

## Methods

### Plant material

This study was based on four Dent (S058, S067, S070, P040) and four Flint (L043, L017, L023, L012) maize inbred lines from the University of Hohenheim, Germany. These inbreds have been selected from an experiment studying the phenotypic reaction of 74 European maize inbreds upon low and high temperature conditions during seedling stage [3]. Out of this set, we selected four heat tolerant (S058, S067, L043, L012) and four heat susceptible (L023, L017, S070, P040) (each two dent and two flint) inbreds for our study.

### Phenotypic evaluation

#### Experimental conditions and assessed traits

Seeds were sown in soil (50% ED73, 50% Mini Tray (Einheitserde- und Humuswerke, Gebr. Patzer GmbH & Co. KG, Sinntal-Altengronau, Germany)) in single pots (9 cm edge length) with *n*=10 replications. The experimental design was a randomized complete block design. The plants were grown at 25°C during a 16h light period and at 20°C during a 8h dark period for a total of three weeks in a walk-in growth chamber (Bronson Incubator Services B.V., Nieuwkuijk, Netherlands). Relative humidity was set to 60%. Photosynthetic active radiation, emitted by fluorescent tubes, was between 270 - 280 μmol *m*^−2^*s*^−1^ in the canopy of the plants to avoid any type of radiation stress, which could be observed with higher light intensities, especially for the Flint germplasm of our study. Watering was conducted every morning to avoid drought stress.

Leaf growth rate was calculated as follows: the length of the fourth leaf from the shoot base to the leaf tip was measured daily for a period of three days during the stage of linear growth. The slope of a linear trendline of leaf length measurements vs. time represented the leaf growth rate. Twenty days after sowing, leaf greenness (SPAD-502, Minolta Corporation, Ramsey, NJ, USA) was assessed as the average value of four readings on the leaf blade of the latest fully developed leaf. Further, the leaf temperature was assessed with an infrared thermometer (Optris LaserSight, Optris GmbH, Berlin, Germany). The plant height from the shoot base to the point where the youngest leaf detached from the older leaf’s sheath and the number of leaves per plant with visible leaf ligule were recorded. A total of 21 days after sowing, shoot dry weight was determined. The above outlined experiment was repeated at two further heat levels, where the temperature was increased after six days to induce heat stress. The mild heat level was at 32°C at day and 27°C at night, the strong heat level was at 38°C at day and 33°C at night. The studied heat levels were chosen such that similar levels of heat stress can be expected in field experiments in Europe.

#### Data analysis

Adjusted entry means for each inbred line - trait - heat level combination were calculated as best linear unbiased estimates using the mixed model

(1) $$ \begin{array}{l}{Y}_{ik}=\mu +{I}_i+{R}_k+{e}_{ik}\kern1em ,\end{array} $$

separately for each heat level, where *Y*_*ik*_ was the observed value for the *i*^*th*^ inbred in the *k*^*th*^ replication, *μ* the general mean *I*_*i*_ the effect of the *i*^*th*^ inbred line, *R*_*k*_ the effect of the *k*^*th*^ replication, and *e*_*ik*_ the residual error. The replications can be seen as a sample of total number of possible replications and, thus, *R*_*k*_ was considered as random factor. The inbred lines were selected specifically for this project and, thus, *I*_*i*_ was considered as a fixed effect.

A principal component (PC) analysis of the adjusted means of the six traits of the eight inbred lines at three heat levels was performed to characterize the overall reaction of the inbred lines at different heat levels. Correlations between the trait means and the first PC (PC1) of the 24 inbred line - heat level combinations were calculated as described by [18]. As a measure of heat susceptibility, the heat susceptibility index (HSI) was defined as the slope of a linear trendline of the loading of an inbred line on PC1 versus the three studied heat levels. Heat susceptible inbred lines were characterized by a high HSI, where heat tolerant inbreds had a low HSI.

To estimate the genotypic variance ${\sigma}_I^2$ and the residual error variance ${\sigma}_e^2$ of the experiment, a further analysis was conducted using model (1) with the genotype effect *I*_*i*_ as random. For each trait, the repeatability *H*^2^ of the results at the three heat levels was calculated using the formula

(2) $$ \begin{array}{l}{H}^2=\frac{\sigma_I^2}{\sigma_I^2+\frac{\sigma_e^2}{n}}\kern1em .\end{array} $$

To check the significance of the effects of the inbred lines, heat levels and the interaction of inbreds and heat levels, a combined model across all heat levels

(3) $$ \begin{array}{l}{Y}_{ijk}=\mu +{I}_i+{H}_j+{(IH)}_{ij}+{R}_{jk}+{e}_{ijk}\kern1em \end{array} $$

was fitted, where *Y*_*ijk*_ was the observed value for the *i*^*th*^ inbred in the *k*^*th*^ replication in the *j*^*th*^ heat level, *H*_*j*_ was the effect of the *j*^*th*^ heat level, (*IH*)_*ij*_ the effect of the interaction between the *i*^*th*^ inbred line and the *j*^*th*^ heat level, *R*_*jk*_ the effect of replication *k* nested in heat level *j*, and *e*_*ijk*_ the residual error. The heat level, inbred line and the interaction effect were set as fixed effects, whereas the replication effect was set as random. All mixed model analyses were performed using the software ASReml [19].

### Transcriptome sequencing

#### Sample preparation and RNA sequencing

At the end of the previously described growing period, leaf samples of the inbred lines were collected at the three heat levels with *n*=10 replications at each of the three heat levels. A sample of about 0.5 cm^2^ was cut from the centre of the latest fully developed leaf of each plant, immediately frozen in liquid nitrogen, and stored at −80°C. The leaf tissue of five replications was pooled to a total of two replications for each genotype - heat level combination to reduce biological variation for the following RNA sequencing. This resulted in a total of 47 samples (the sample for one replication of a genotype - heat level combination was missing). Total RNA was isolated using the RNeasy Plant Kit (Qiagen, Hilden, Germany). RNA quantity was assessed and quality control was performed using a Qubit fluorometer (Life Technologies, Darmstadt, Germany) and the 2100 Bioanalyzer (Agilent Technologies, Böblingen, Germany). DNA was removed using the TURBO DNA free Kit (Ambion, Kaufungen, Germany) and the solution was purified using the RNeasy ^*Ⓡ*^ MinElute ^*Ⓡ*^ Cleanup spin columns (Qiagen). rRNA was depleted prior to sequencing using the RiboMinus™Plant Kit (Invitrogen, Life Technologies, Darmstadt, Germany). Library preparation and RNA sequencing were performed at the Max Planck Genome Centre Cologne using an Illumina HiSeq2000 sequencing machine (Illumina, Inc., San Diego, CA USA). The 47 samples were combined to eight 100-bp single-end Illumina sequencing libraries with each six (one with five) individually barcoded samples. Each library was sequenced on one lane of the sequencing machine.

#### Data analysis

Outcoming single-end sequence reads were cleaned for reads containing primer or adaptor sequences. Sequencing reads with more than 30% of bases with a Phred quality score of ≤20 were excluded from the following analyses (cf. [20]). High quality reads were aligned to the B73 reference sequence (AGPv3 release 20) using TopHat (Version 2.0.3, [21]). We used the R package easyRNASeq (Version 1.6.2, [22]) to filter the aligned reads for protein-coding genes located on the nuclear chromosomes and counted transcript reads per gene model in the 47 samples. As there is no purpose in analysing genes, which are not expressed at a reasonable level in none of the inbred line - heat level combinations, we excluded poorly expressed genes which did not show at least two counts per million reads in at least two samples (cf. [23]). The biological coefficient of variation (BCV) was calculated according to [24] from the square root of the common dispersion using the R package EdgeR (Version 3.2.4, [23]). The easyRNASeq table of counts was subject to a PC analysis to assess transcriptomic variation in the 47 samples and identify clustering of inbred lines, heat levels, and heterotic pools using the R package DeSeq (Version 1.10.1, [25]).

To identify first, genes involved in heat response and second, heat tolerance related genes, we selected three sets of candidate genes. (i) Genes with differential regulation upon increasing heat stress, where the eight inbred lines were considered as replications of one average genotype. These genes are designated in the following as overall heat responsive genes. (ii) Genes with differential expression in every single inbred line, where the individual inbreds were considered and the number of overlapping differentially expressed genes between the inbreds was assessed. These genes are designated in the following as common heat responsive genes. (iii) Genes, where differential regulation upon increasing heat stress was a function of the phenotypically assessed heat tolerance of each inbred line. These genes are designated in the following as heat tolerance genes.

For establishing the set of overall heat responsive genes, expression of each gene across all inbred lines at a heat level was explained by the metric value of the respective heat level using the linear regression model in EdgeR:

(4) $$ \begin{array}{l}{Y}_{ijk}=\mu +{x}_j\beta +{e}_{ijk}\kern1em ,\end{array} $$

where *Y*_*ijk*_ was the expression of the respective gene of inbred *i* at heat level *j* in replication *k*. *μ* was the y-intercept and *β* the slope of the linear regression respectively. *x*_*j*_ defined the *j*^*th*^ heat level, where the heat levels 25°C and 38°C were assigned the metric values 0 and 1. Correspondingly the 32°C heat level was assigned *x*=7/13. *e*_*ijk*_ was the residual error term. *β* was estimated to obtain the expression change across all inbred - replication combinations across the three heat levels. The data samples were normalized with EdgeR’s internal normalization procedure for library size and dispersions between biological replications were calculated genewisely.

In this study, genes with a false discovery rate (FDR) [26] of <0.05 and |*log*_2_(*β*)|>2 were considered as significantly differently expressed genes. MAPMAN (Version 3.6.0RC1, [27]) was used to classify the overall heat responsive genes by biological function and to graphically illustrate them in a custom created overview of involved molecular processes (mapping file version ZM_B73_5b_FGS_cds_2012). For the same set of genes, information on genome position and gene description (www.uniprot.org) was accessed via the R package bioMart (Version 2.16.0, [28]). Gene ontology (GO) terms were assigned to each of the overall heat responsive genes and a GO term enrichment analysis was carried out [29] using the Zea mays ssp maize genome locus reference (maizesequence.org). To determine significantly enriched GO terms between the heat responsive genes within the RNA-Seq approach and the reference a hypergeometric test with FDR <0.05 was applied for the upregulated and downregulated genes, separately.

In a next step, we identified heat responsive genes for each inbred line by using the model:

(5) $$ \begin{array}{l}{Y}_{ijk}=\mu +{x}_j{\gamma}_i+{e}_{ijk}\kern1em ,\end{array} $$

to calculated the expression change of each gene for one inbred line. In this model, *γ*_*i*_ represented the slope of the linear regression, and thus the expression change, for each inbred line *i*. Genes with an FDR <0.05 and |*log*_2_(*γ*)|>2 were considered as significantly differently expressed genes for each inbred line. The overlapping differentially expressed genes among all inbred lines was examined to define the set of common heat responsive genes.

The set of heat tolerance genes was established applying the following linear model,

(6) $$ \begin{array}{l}{\gamma}_i=\mu +{h}_i\delta +{e}_i\kern1em ,\end{array} $$

where *γ*_*i*_ was the expression change for the *i*^*th*^ inbred, estimated with model (5) and *h*_*i*_ the HSI of the *i*^*th*^ inbred. *δ* was the slope of the linear regression for the respective gene across all inbreds which represented the magnitude of differential regulation between heat tolerant and heat susceptible inbred lines. The heat tolerance genes showed a significant (P <0.05) association between *γ*_*i*_ and the HSI and a slope of |*δ*|>2 across inbred lines. The heat tolerance genes were included in a heatmap with log_2_ fold change (log _2_*γ*_*i*_) of each inbred line over heat levels 0, 7/13 and 1, i.e. 25°C, 32°C and 38°C, where genes were clustered by their differential reaction across the inbreds lines. Information on biological processes of the heat tolerance genes was obtained using the R package biomaRt with dataset zmays_eg_gene and from MaizeGDB (www.maizegdb.org).

To validate RNA sequencing results, quantitative real-time PCR (qRT-PCR) was conducted using DyNAmo SYBR Green 2-Step qRT-PCR Kit (Thermo Scientific, Bremen, Germany). Primers were developed using Primer3web interface (primer3.ut.ee, Version 4.0.0, [30]) for 11 genes, randomly selected from the total set of detected genes, excluding poorly expressed genes, and for *Actin*1 (gene GRMZM2G126010) as a reference gene. RNA extraction and DNAse treatment were carried out as described previously and the RNA of ten replications was pooled. A total of 24 RNA samples (inbred line - heat level combinations) each with 2.5 *μg* was reversely transcribed using SuperScript™First-Strand Synthesis System for RT-PCR (Invitrogen, Life Technologies). The PCR protocol was replicated three times as follows: Initially 96°C for 2 minutes was followed by 40 cycles of each 96°C for 30 seconds, 55°C for 45 seconds and 72°C for 90 seconds. The last step at 72°C lasted 5 minutes. To determine the correlation of sequencing and qRT-PCR, the relative log_2_ fold expression changes for the mentioned 11 genes to *Actin*1 was calculated for the data obtained by sequencing and by qRT-PCR for each inbred line - heat level combination according to [31].

## Results

**Figure 1:**
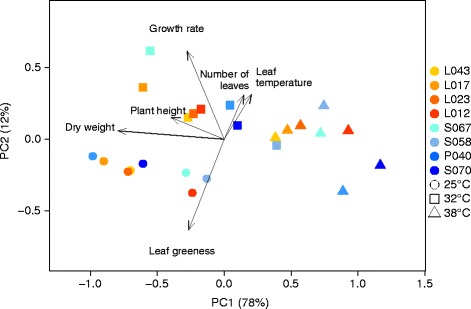
**PCA of phenotypic data.** Biplot of principal component (PC) analysis of inbred - heat level means for six traits of eight inbred lines examined at three heat levels. Numbers in parentheses represent the percentage of total variance explained by the first and second PC. Yellow and red colors represent Flint, and blue represents Dent inbred lines. The shapes (circle, square, triangle) represent the studied heat levels (25, 32 and 38°C).

**Figure 2:**
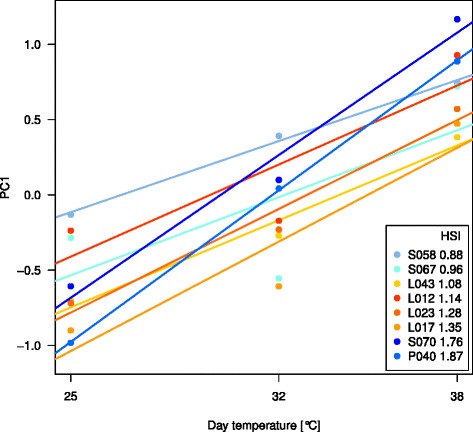
**Linear regression of PC1.** First PC of the PC analysis with six phenotypic traits of each inbred line plotted over three heat levels. The slope of the linear regression represents the heat susceptibility index (HSI).

Repeatabilities of the assessed traits at each of the three studied heat levels were high with values between 0.59 and 0.93 (Table 1). All measured traits were monotonic increasing or decreasing with increasing heat level, except the leaf elongation rate, which showed a maximum at 32°C. Effects of inbred lines and heat levels as well as the interaction between both were significant (P <0.001) for all studied traits. The first two PCs of the PC analysis of the phenotypic data (Figure 1) explained 78 and 12% of the total variance of the inbred line-by-heat level means (Additional file 1). PC1 correlated significantly (P <0.01) with all measured traits with |*r*| between 0.59 and 0.99. We observed a clustering of the samples with respect to the heat levels, whereas no clustering of inbred lines or heterotic pools was observed. The HSI ranked the inbred lines in the order from tolerant to susceptible: S058, S067, L043, L012, L023, L017, S070 and P040 (Figure 2). Dent inbreds were the two most heat tolerant and the two most heat susceptible genotypes.

**Table 1:** **Repeatability, mean trait value, and correlation of traits with PC1 across eight inbred lines examined at three heat levels**

	**Repeatability at**			**Mean value at**			**Correlation**
**Trait**	**25°C**	**32°C**	**38°C**	**25°C**	**32°C**	**38°C**	**with PC1**
Growth rate [cm/hour]	0.80	0.85	0.88	0.24	0.29	0.19	-0.67^***^
Dry weight [g]	0.71	0.77	0.93	2.02	1.55	0.62	-0.99^***^
Plant height [cm]	0.78	0.88	0.89	21.9	20.6	12.5	-0.93^***^
Number of leaves	0.91	0.90	0.89	3.5	4.4	4.6	0.59^**^
Leaf temperature [°C]	0.71	0.59	0.89	24.7	31.8	36.4	0.81^***^
Leaf greenness [SPAD value]	0.90	0.90	0.93	47.1	34.2	29.4	-0.68^***^

^**^Significant with P <0.01, ^***^Significant with P <0.001.

**Figure 3:**
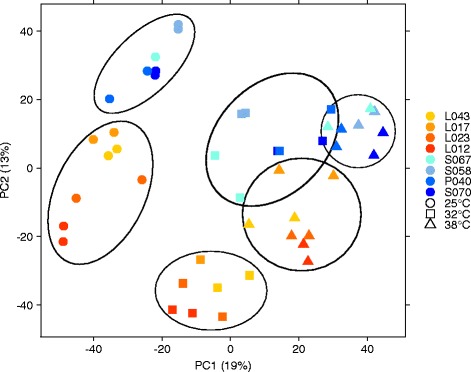
**PCA of transcriptomic data.** Principal component (PC) analysis from *DeSeq* of the gene counts of eight inbred lines examined at three heat levels and two replications. Numbers in parentheses represent the percentage of total variance explained by the first and second PC. Yellow and red represent Flint, and blue represents Dent inbred lines. The shapes (circle, square, triangle) represent the studied heat levels. Circles represent the six pool-by-heat level clusters.

RNA sequencing resulted in a total of 1,461,089,891 single end sequence reads across all 47 samples. The BCV was 0.26 in this experiment. A total of 19 and 13% of the variation of the high-quality protein-coding chromosomal transcripts was explained by the first two PCs of the PC analysis of the transcriptomic data (Figure 3). PC1 and PC2 separated six clusters, where PC1 separated the three heat levels and PC2 separated mainly the pools Flint and Dent. We identified 17,905 genes with at least two counts per million in at least two samples. A total of 567,485,727 transcript reads accounting for previously described genes were used for the following analyses.

**Figure 4:**
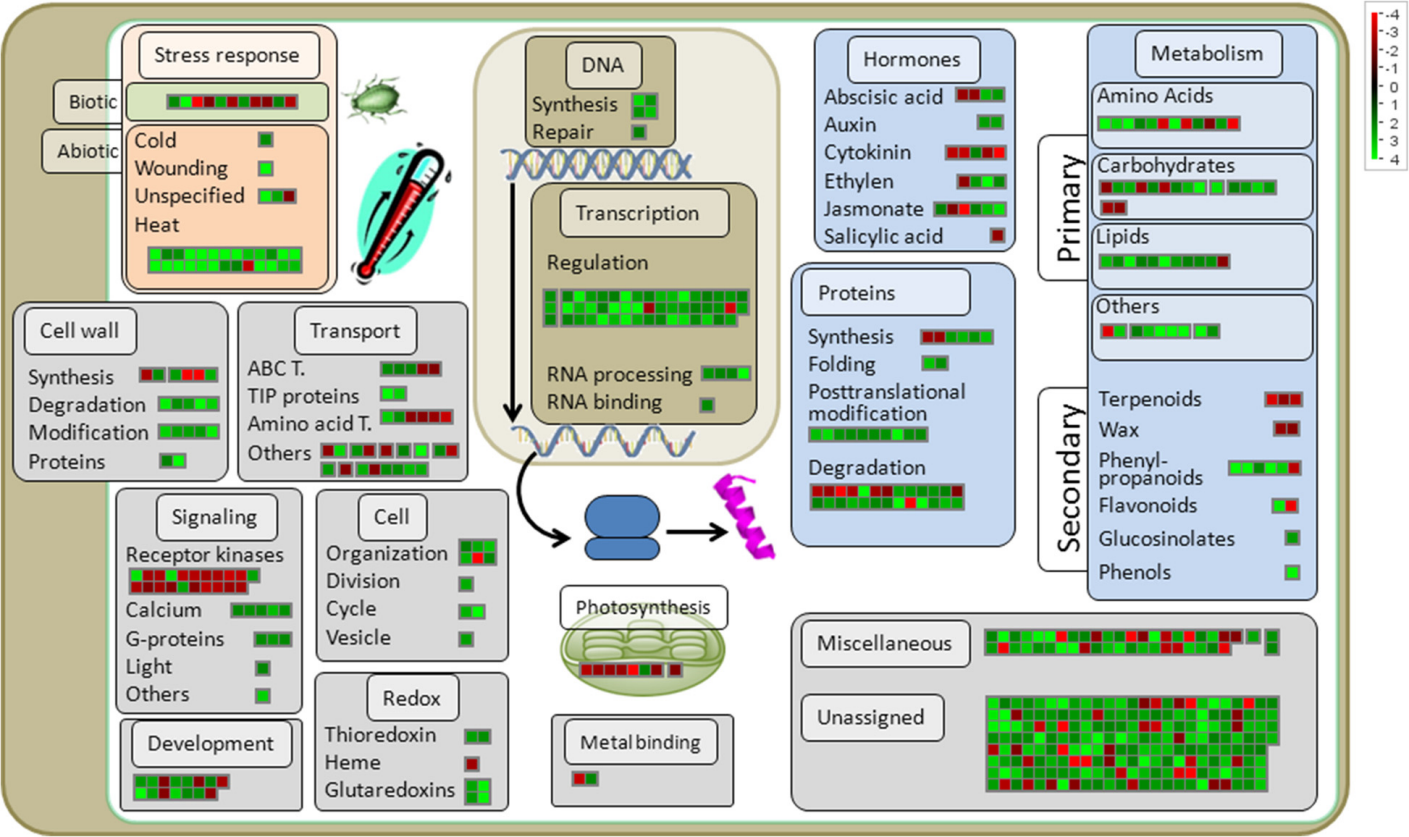
**Heat responsive genes.** Biological functions of overall heat responsive genes (FDR <0.05 and |*log*_2_(*β*)|>2 with increasing heat levels across all inbred lines). Colours represent log_2_ fold changes higher (green) and lower (red) than 0.

**Figure 5:**
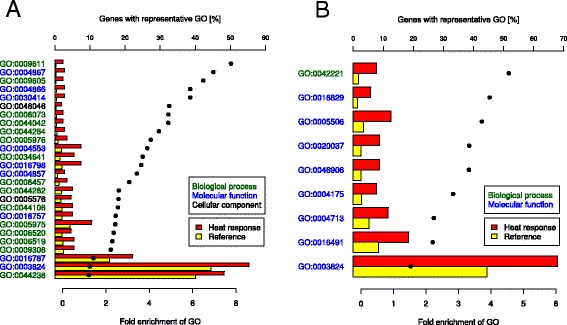
**Enriched GO terms in the set of heat responsive genes.** Significantly (FDR <0.05) enriched GO terms for biological process, molecular function, and cellular component in the upregulated **(A)** and downregulated **(B)** heat responsive genes are plotted according to increasing enrichment (dots) of the percentage of genes in the RNA-Seq gene set (red bars) compared to that of the maize reference set (yellow bars). Mentioned GO are described in Additional file 4.

We identified across all inbred lines 607 overall heat responsive genes, of which 460 were up- and 147 downregulated, when considering increasing heat levels. A total of 594 of these genes a biological function could be assigned by MAPMAN (Figure 4, Additional files 2 and 3). Our data indicated the involvement of 53 types of biological functions in the overall heat responsive genes. The biological function of heat response, containing 14 heat shock genes, included exclusively upregulated genes (Additional file 3). Furthermore, genes involved in the regulation of transcription, DNA replication, and posttranslational modification of proteins were, except for two genes, upregulated with increasing heat levels. The GO terms analysis resulted in 26 enriched GO terms in the upregulated overall heat responsive genes and 9 enriched GO terms in the downregulated heat responsive genes (Additional file 4) with an up to 8 fold GO enrichment (Figure 5). The over-represented cellular component GOs were related to the apoplast and the extracellular region (Additional file 4). Within the GOs associated with biological processes, responses to external stimulus, the amino acid and protein metabolism, as well as to the carbohydrate metabolism were enriched. Concerning the GOs related to molecular function, these can be roughly grouped to catalytic activities, enzyme regulation and tetrapyrrole binding.

The number of highly differentially regulated genes for each inbred line (identified using model (5)) was between 227 and 695 (Table 2), where the number of upregulated genes was generally higher than the number of downregulated genes. The number of genes that were commonly differentially expressed in all inbred lines was 14, where 7 genes were upregulated and 7 genes were downregulated (Table 3). The 7 commonly upregulated genes included three heat shock genes and two genes previously characterized as being heat responsive. The 7 commonly downregulated genes, in contrast, did not include genes which were described as heat responsive, but included diverse classes of genes.

**Table 2:** **Number of heat responsive genes for each inbred line, identified with model (** 5 **), which were differently expressed (FDR** ***<0.05*** **and expression change** **|** ***log***_**2**_ ***(γ)|>2*** **) with increasing heat levels and the overlapping genes between the eight inbred lines (common heat responsive genes), between Flint and Dent inbreds, respectively and between heat tolerant and susceptible inbreds, respectively**

**Inbred line**	**S058**	**S067**	**L043**	**L012**	**L023**	**L017**	**S070**	**P040**	**Overlap**	**Flint**	**Dent**	**Tolerant** ^*^	**Susceptible** ^**^
Upregulated	395	284	133	515	225	290	289	177	7	13	28	21	17
Downregulated	248	130	94	180	108	152	202	141	7	11	22	17	17

^*^Tolerant inbreds S058, S067, L043, L012.

^**^Susceptible inbreds L023, L017, S070, P040.

**Table 3:** **List of the common heat responsive genes, which were differentially expressed (FDR** ***<0.05*** **and** ***|log***_***2***_ ***(γ)|>2*** **) with increasing heat levels in each inbred line, with mean log**_**2**_ **(** ***γ*** **), the mean expression change of the respective gene with increasing heat levels across all inbred lines**

**Gene**	**Mean log**_**2**_ **(** ***γ*** **)**	**Gene description**
GRMZM5G833699	8.29	Heat shock protein
GRMZM2G149647	8.26	Heat shock protein 26; Small heat shock protein
GRMZM2G366532	7.51	Heat response
GRMZM2G007729	5.92	Heat response
GRMZM2G158394	4.92	Extracellular ribonuclease
AC209784.3_FG007	4.10	Heat shock protein 70, MreB/Mbl protein
GRMZM2G111014	2.41	DNA.synthesis/chromatin structure
GRMZM2G057611	-2.81	Peptides transport protein
GRMZM2G147819	-3.15	Uncharacterized protein
GRMZM2G439195	-3.27	Nicotianamine synthase (metal handling)
GRMZM2G009189	-4.26	Uncharacterized protein
GRMZM2G114588	-4.34	Isoflavone reductase (secondary metabolism)
GRMZM2G125314	-4.99	LOL3 (protein.degradation)
GRMZM2G173710	-6.77	Cytokinin, signal transduction

**Figure 6:**
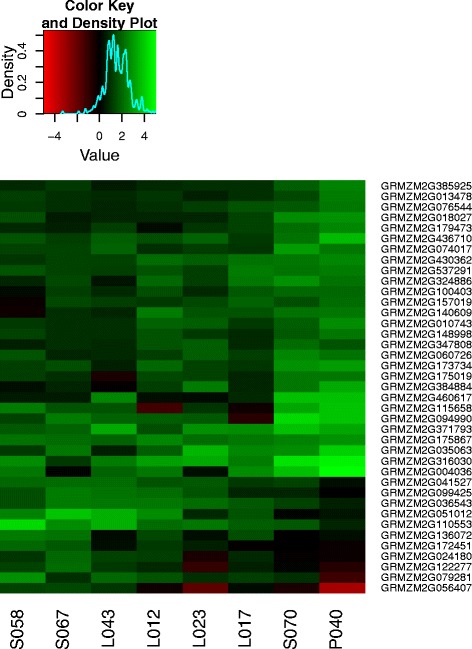
**Heat tolerance genes.** Differential regulation of the heat tolerance genes, which have been identified with significant (P <0.05) linear association between *γ* and the heat susceptibility index with a slope of |*δ*|>2, in eight inbred lines. Colours represent log_2_ upregulation (green) and downregulation (red) with increasing heat levels.

By explaining the gene expression change of each inbred line by its phenotypic HSI, we identified 39 heat tolerance genes (Table 4). These heat tolerance genes were divided into two major clusters of genes by their expression in heat susceptible inbreds (Figure 6). The first class included 28 genes with strong upregulation of gene expression with increasing heat levels in heat susceptible inbreds compared to heat tolerant inbreds, whereas the second class of 11 genes showed the contrary regulation pattern. Seven of the heat tolerance genes were also overall heat responsive genes. The heat tolerance genes were of a variety of biological functions. Amongst others, we found that the heat tolerance genes have a predicted biological function of protein folding and biosynthesis, cell wall modification, and calcium signalling.

**Table 4:** **List of the heat tolerance genes, with significant (P** ***<0.05*** **) association between** ***γ*** **and the heat susceptibility index and a slope of** ***|δ|>2*** **across inbred lines**

**Gene identifier**	***δ***	**Biological function (Possible function found for** ***Oryza sativa L.*** ** or** ***Arabidopsis thaliana L.*** ** orthologs)**
GRMZM2G385925	3.39	Kinesin heavy chain-like protein
GRMZM2G013478	2.41	Nucleoside diphosphate kinase
GRMZM2G076544	2.72	Peptidyl-prolyl cis-trans isomerase
GRMZM2G018027	5.62	OXIDATIVE STRESS 3 (ATOXS3, *A. thaliana* best hit)
GRMZM2G179473	4.28	Inositol-tetrakisphosphate 1-kinase 3
GRMZM2G436710	7.09	Unknown
GRMZM2G074017	4.43	ATPase inhibitor
GRMZM2G430362	3.71	ATP-dependent RNA helicase SUV3
GRMZM2G537291	2.82	Uncharacterized protein
GRMZM2G324886	4.31	Unknown
GRMZM2G100403	2.80	Ribosomal protein L4/L1 family
GRMZM2G157019	2.16	Nucleosome/chromatin assembly factor A
GRMZM2G140609	3.47	40S ribosomal protein S23
GRMZM2G010743	3.59	Tim17/Tim22/Tim23/Pmp24 family
GRMZM2G148998	2.23	Unknown
GRMZM2G347808	2.02	RNA cap guanine-N2 methyltransferase
GRMZM2G060726	3.53	Transcriptional regulator
GRMZM2G173734	5.74	Protein phosphatase
GRMZM2G175019	3.39	Unknown
GRMZM2G384884	7.31	Cytochrome P450 (Phenol stress [32])
GRMZM2G460617	12.88	Unknown
GRMZM2G115658	10.01	Unknown
GRMZM2G094990	14.66	Rare lipoprotein A (RlpA)-like double-psi beta-barrel
GRMZM2G371793	7.58	Uncharacterized protein
GRMZM2G175867	2.47	Putative DEAD-box ATP-dependent RNA helicase family protein
GRMZM2G035063	10.50	Chaperonin (Heat stress [33])
GRMZM2G316030	22.34	UDP-glucoronosyl and UDP-glucosyl transferase (Salinity stress [34])
GRMZM2G004036	35.26	Short-chain dehydrogenase (Metals and oxidizing chemicals and reduction of superoxide radicals [35], *Striga hermonthica (Del.) Benth* infection [36], defoliation [37])
GRMZM2G041527	-2.84	Ribonucleases P/MRP protein subunit POP1
GRMZM2G099425	-3.44	Calcium-dependent protein kinase, isoform AK1
GRMZM2G036543	-2.81	Histidine biosynthesis protein
GRMZM2G051012	-9.69	Unknown
GRMZM2G110553	-11.06	Unknown
GRMZM2G136072	-3.29	Glyoxylate reductase
GRMZM2G172451	-2.56	Plant organelle RNA recognition domain
GRMZM2G024180	-2.19	RNI-like superfamily protein
GRMZM2G122277	-2.40	Cellulose synthase (locus rs129668732) (Salt stress [38])
GRMZM2G079281	-3.99	Unknown
GRMZM2G056407	-2.16	MYB family transcription factor

Validation of the sequencing data by qRT-PCR resulted in a highly significant correlation of p <0.001, r =0.68 between the relative expression changes of 11 genes for 24 inbred line - by heat level combinations, obtained by RNA sequencing and by qRT-PCR (Additional file 5).

## Discussion

### Phenotypic variation for heat tolerance of European Flint and Dent maize inbred lines

We observed a high repeatability for all evaluated phenotypic traits at the three examined heat levels (Table 1). This was in accordance with results of [39], who detected similar levels of repeatability for the traits leaf greenness and plant dry weight under optimal (27/25°C) and chilling conditions (16/13°C) in a set of Flint and Dent inbred lines in growth chamber experiments. The true trait means for the genotypes at the studied heat levels could, thus, be estimated reliably in our study and therewith are a good basis for the following genome-wide expression profiling experiment.

The low and strong reduction of mean dry weight at 32 and 38°C, respectively, compared to 25°C, indicates that we were successful in setting the appropriate temperatures of the medium and the strong heat level. The trait means across inbred lines for leaf greenness and dry weight per plant showed different alteration with increasing heat levels, depending on the severity of heat stress. Compared to 25°C, at the medium heat level (32°C), the mean leaf greenness across all inbreds was reduced notably, where the mean dry weight was only slightly decreased. At the high heat level (38°C) in turn, leaf greenness did not show further notable decrease in comparison with 32°C, where dry weight was decreased substantially (Table 1). Chlorophyll content, which is correlated with leaf greenness [40], was reduced in wheat at high temperature (38°C in average) compared to control temperature (26°C in average) in a study of [41]. Generally, growth reduction in plants upon high temperature stress may be due to reduced photosynthesis, which is associated with leaf greenness, caused by an injury of the photosynthetic system [42]. Fokar 1998, [41] found a negative (although not significant, P ≥0.05) association between chlorophyll retention and grain filling, as a measure for plant performance, and stated that grain filling could even be promoted by fast leaf senescence i.e. leaf greenness reduction, as metabolites might be transported from senescent tissue to the grain. This effect could be similar in our study, where at 32°C, where leaf greenness was highly reduced, metabolites could sustain plant growth and development. Another effect sustaining plant growth is the increased development speed at increased heat level, which was observed from the increased number of leaves per plant at heat stress. The increased growth rate at 32°C compared to the lower as well as higher temperature regime was probably due to this increased speed of development at increased temperature, where plants were still not greatly damaged by heat stress.

To obtain a description of total plant performance across the three examined heat levels, we performed a PC analysis integrating all observed traits, i.e. leaf growth rate, shoot dry weight, plant height, the number of leaves, leaf temperature, and leaf greenness. PC1 explained with 78% a high proportion of the total variance and was correlated significantly (*α*<0.01) with each observed trait (Table 1). Therewith, in our study, PC1 was sufficient as a unique integrative trait to explain plant performance. In order to quantify heat tolerance in maize seedlings, several morphological and physiological traits were studied by [43]. In this paper, the shoot fresh and dry mass, shoot length, leaf area, growth rate, increase in leaf area, and the assimilation rate were used as single traits to quantify the reaction of maize seedlings upon strong heat stress (38°C day temperature). Our integrative plant performance trait, i.e. PC1, has the advantage that it represents each of the observed traits and gives a broad picture of plant performance under stress conditions with one single value for each genotype - heat level combination.

To quantify heat tolerance on a multi-trait level, the HSI was calculated as the slope of a linear regression of PC1 over the three examined heat levels. We observed a strong difference in heat tolerance between the eight inbred lines with HSI ranging from 0.88 up to 1.87 (Figure 2). Two of the inbred lines (S070 and P040) showed a high HSI (Figure 2) and were therewith considered as heat susceptible. In contrast, the other six inbreds showed lower HSIs and were therewith considered as more heat tolerant than the before mentioned two inbreds. This finding was associated with a significant (*α*<0.001) inbred line - heat level interaction, which was observed for all examined traits. We have, thus, a very divers set of inbreds, which indicates that our study is appropriate to investigate heat response and elucidate the molecular mechanisms of heat tolerance in diverse genetic backgrounds.

Despite observing diverse heat tolerance reactions for the eight examined inbreds, we found that neither Flint nor Dent inbreds showed systematically higher or lower heat tolerance (Figure 1). The same trend was observed for the adaptation to low temperatures, where [39] showed that both European Flints and European Dents showed chilling tolerance. Besides this finding, we observed that Dent inbreds were the most heat tolerant and the most heat susceptible inbreds, suggesting that the Dent pool shows more variability in terms of tolerance to high temperature during seedling stage.

### Transcriptomic variation

The validation of the transcriptome sequencing results by qRT-PCR resulted in an r of 0.68 (Additional file 5), and therewith were in the order of magnitude of results of another RNASeq study in maize [31]. This finding indicated that our RNA sequencing results were reliable.

We observed in our study that the biological coefficient of variation (BCV) of the transcriptomic data across all observed genes and inbred - heat level combinations was 0.26 and was therewith in the range of previously reported values [44].

The number of read counts per sample in our study was between 5 and 19 million (Table 5). The mean library size was in accordance with results of [31], who observed a median library size of 7.6 million reads. In the current study, we observed a remarkable variation of library size between the three examined heat levels. Tarazona 2011, [45] advised a balanced sequencing depth between conditions for differential expression analyses to support accurate statistical analyses. As the number of read counts was at the minimum 5 million for one sample in our study, however, we do not expect an impact on the power to detect differentially expressed genes.

**Table 5:** **Mean number of high quality reads aligned to protein-coding chromosomal genes of RNA sequencing results of eight inbred lines growing at three heat levels**

**Inbred line**	**25°C**	**32°C**	**38°C**
L012	12,339,774	14,851,918	13,329,198
L017	13,769,342	8,072,699	8,376,793
L023	11,568,570	11,703,940	8,505,396
L043	19,314,142	11,881,390	10,475,758
P040	15,637,596	9,184,952	7,922,566
S058	14,812,251	14,107,242	7,922,258
S067	19,073,758	12,920,434	4,874,014
S070	15,755,252	11,228,139	10,151,830

The genes, for which the detected transcript reads accounted, showed a strong expression variation between inbred lines (Figure 3). Consistent with the PCA of the phenotypic data (Figure 1), we observed a clustering of the heat levels in the PCA of the transcriptomic data. Furthermore, we observed a clustering of the inbreds with respect to transcriptomic variation according to their heterotic pool assignment, which was not observed for the phenotypic data. This finding was in good agreement to the findings of clustering of heterotic groups with respect to genotypic variation in previous studies [39,46,47]. The differentiation we observed based on the genome-wide expression data between the Flint and Dent pool was stronger than the differentiation between heat tolerant and heat susceptible inbreds. This finding can be explained by the separate breeding history of Flints and Dents, based on their introduction into Europe from the Americas [48] and the pools show, thus, strong genetic and transcriptomic differences.

### Approaches to identify heat responsive genes

In this study, two different approaches for identifying heat responsive genes were applied in order to cover the different aspects. Firstly, we identified overall heat responsive genes as genes with differential regulation upon increasing heat levels, where the eight inbreds were considered as replications of one average genotype. The set of overall heat responsive genes can help to understand molecular defence mechanisms against heat stress of maize. This set represents genes showing the general response of temperate maize to heat stress and may not be essential for each inbred lines’ heat response, but include all strategies to cope with heat stress used by the studied maize inbreds. In our study a broad set of maize inbreds were studied, whereas in previous papers mostly two contrasting genotypes were included to study the transcriptomic response upon abiotic stress. Therefore we expect that based on our results generally applicable statements on transcriptomic heat response of European Flint and Dent inbreds are possible.

In addition to the overall heat responsive genes, we identified the common heat responsive genes by overlapping the heat responsive genes of each inbred line. These genes account for heat responsive mechanisms shared by all inbred lines. The common heat responsive genes represent a small set of genes, which are, as they are differentially expressed in each inbred line likewise, absolutely necessary, i.e. indispensable key genes for the heat response as discussed for drought stress in maize by [49].

### Molecular response of temperate maize upon increasing heat levels

In the set of 607 overall heat responsive genes, three GO terms, associated with the response to external stress (GO:0009611, GO:0009605, GO:0042221), were enriched (Figure 5 and Additional file 4). This suggests a strong connection of the response to heat stress with other types of external stress response.

Furthermore, we observed an upregulation upon increasing heat levels of seven calcium-dependent signalling genes (Figure 4). As membrane fluidity is increased with increasing temperature, this results in an increased calcium-ion influx in the cells [9], serving as messenger for stress signalling [50]. Our results are in accordance with the previously reported finding that calcium-dependent signalling genes play essential roles in plant response to abiotic stress [51].

Stress signalling pathways, e.g. calcium signalling, in turn, trigger the regulation of transcriptional factors [52]. Transcription regulation genes were the most prominent group of heat responsive genes in our study with 40 upregulated genes. They have the potential to activate further stress responsive mechanisms to re-establish cell homeostasis, to protect, as well as repair proteins and membranes [52].

Coping with the damages produced by oxidative stress is viable for plant survival at heat stress. The binding of tetrapyrrole, which was found to be associated with oxidative stress and cell death in plants [53], is associated with three GO terms (GO:0005506, GO:0020037, GO:0046906), enriched in the heat responsive gene set (Figure 5). We observed, further, an upregulation of six antioxidant genes (Thioredoxins and Glutaredoxins) and six cytochrome P450 related genes in the set of overall heat responsive genes (Figure 4). These genes are known to be involved in the antioxidant defence of plants [54] and act in the detoxification of damages due to oxidative stress [55,56].

Further we found an increased expression of 11 heat responsive genes associated with the lipid metabolism. Plants try to change membrane composition as an adaptive mechanism to heat stress [9,57]. The identified lipid metabolism genes could be involved in phospholipid changes of the membrane composition to protect and recover damaged cell membranes. However this requires further research.

We observed that with 44 a high number of the 607 overall heat responsive genes were involved in the protein metabolism. Furthermore, we identified 14 heat shock genes, which act as chaperones and are involved in protein-folding [52]. This finding was supported by 5 GO terms associated with protein folding (GO:0006457) and amino acid metabolism (GO:0044106, GO:0006520, GO:0006519, GO:0009308), which were enriched in the upregulated heat responsive genes (Figure 5). This illustrates that protection of proteins against oxidative stress is another key component of the response to heat stress in maize.

Several GO terms were enriched in the upregulated heat responsive genes (Additional file 4), which are associated with carbohydrate metabolism (GO:0006073, GO:0044042, GO:0044264, GO:0005976, GO:0044262, GO:0005975) and could play a role in a modification of starch synthesis at heat stress.

The set of overall heat responsive genes illustrates that heat stress response in temperate maize involves a multitude of biological processes (Figure 4). The enrichment of GO terms in the heat responsive genes (Figure 5), which revealed the involvement of numerous biological functions and molecular processes in the heat response was in agreement with this statement.

The overlap of the heat responsive genes of each inbred between inbred lines, designated as the common heat responsive genes (Table 2), was very small, representing 1% total of genes detected as heat responsive in one of the eight inbreds. Our finding indicated that individual inbred lines developed different genetic mechanisms in response to environmental stress, which overlap only to a small degree between genotypes. Therefore it is advisable to include a variety of genotypes with different genetic backgrounds and origin in abiotic stress expression studies in order to combine different genetic strategies to cope with heat stress.

### Identification of heat tolerance genes

We used a new approach to identify heat tolerance genes, which is characterized by the inclusion of phenotypic and environmental variation in the statistical analysis. The traditional approach to select stress tolerance candidate genes is to compare two groups of genotypes with contrasting stress tolerance, as outlined in several studies, discussed previously in this paper in the context of heat response. The inclusion of a diverse set of inbred lines with high variation for heat tolerance in our study, has the advantages of, first, considering the continuous distribution of the values of quantitative traits, and second, considering phenotypically intermediate genotypes without focussing only on the extremes.

Further, we included a linear regression model across three heat levels to identify differentially expressed genes over a temperature gradient, which is rarely used in other abiotic stress tolerance studies. [49] considered three levels of stress intensity (drought), but compared gene expression of pairs of stress levels instead of evaluating linear dependency of gene expression across stress conditions. This results in an increased number of statistical tests. Our approach consisted in one statistical test, including all stress levels, which leads to a reduction of the multiple-test problem. Furthermore, our approach has the advantage of considering the effect of a linear increase in temperature more independently of the actual studied heat levels. We could, thus, identify heat tolerance candidate genes for a stress intensity range between the examined heat levels in our study, i.e. from 25°C to 38°C. These two particularities can be reasons that none of the heat tolerance genes, identified in our study, was previously described in literature to be involved in heat tolerance in maize.

In this study, the p-value, which states the significance of the linear dependency between the expression change for each inbred line and the HSI of the respective inbred, was not adjusted for multiple testing to not lower even more the low power to detect heat tolerance genes. This low power comes from the consideration of phenotypic variation for heat tolerance in the gene identification method, which is comparable with an association mapping approach. In typical association mapping studies in maize, the number of genotypes in a population ranges between around 100 and 500 individuals [58]. We conclude that, in a study to identify candidate genes using transcriptome profiling, which includes phenotypic variation, it is indispensable to use a higher number of genotypes, comparable to those of association mapping studies. Nevertheless, for reasons of completeness, we discuss the identified heat tolerance genes.

The heat tolerance genes identified in this study were upregulated in most of the inbred lines and there was rarely downregulation in one of the inbreds (Figure 6). This indicated that genes which are differentially regulated between inbred lines based on phenotypic heat tolerance are typically genes, which are generally upregulated with increasing heat levels. Genes, which are downregulated with increasing heat levels are typically not differentially regulated between inbred lines. This may be partly explained due to the finding that, in general, more genes are upregulated than downregulated at heat stress, as it comes obvious from the set of overall heat responsive genes (Figure 4). Nevertheless, there must be a further, still elusive, physiological or statistical explanation for the almost absence of downregulated genes in the set of heat tolerance genes.

For 6 of the total of 39 heat tolerance genes, earlier studies indicated a mechanistic involvement in different abiotic stress responses including salt, heat and oxidative stress. This finding could be explained by interconnection between the molecular responses to different kinds of abiotic stresses, which similarly produce osmotic and oxidative stress on the cellular level [52].

To further validate, if the identified 39 heat tolerance genes explain phenotypic variation for heat tolerance, further studies have to be performed e.g. with segregating populations derived from crosses of heat tolerant and heat susceptible inbred lines presented in this study. In such experiments with a similar experimental design as the present study, the expression change with increasing heat levels of the 39 heat tolerance candidate genes could be detected using RNA sequencing or qRT-PCR and correlated with the phenotypic heat tolerance of each genotype from the segregating populations. Another validation approach could be to overexpress or inhibited the expression of the heat tolerance candidate genes in selected inbred lines. Comparing the phenotypic heat tolerance of the modified genotype with non-transformed genotypes can evidence a possible heat tolerance function of the respective gene. In a subsequent experiment, thus transformed genotypes and previously mentioned segregating populations could be tested at heat stress conditions in field experiments to examine if the selected candidate genes have a heat tolerance function in a natural environment as well as in the adult stage. Validated genes could then be used in a molecular breeding approach, in order to obtain heat tolerant varieties.

## Conclusion

In this study, we found a high variation for heat tolerance during seedling stage in a set of European maize inbred lines, which is not dependent on heterotic pools, but comes with different molecular strategies of single inbred lines to cope with increasing heat levels. We could, further, support and expand knowledge of the heat response pathways in maize and plants in general (Figure 4). Finally, we identified 39 heat tolerance candidate genes (Figure 6), whose molecular function for heat tolerance and adaptation is unknown and should be clarified using functional studies. We suggest further the performance of transcriptome profiling experiments with populations of inbred genotypes derived from biparental crosses in order to improve the power to detect significance of detected heat tolerance genes.

## Availability of supporting data

The data sets supporting the results of this article are included within the article and its additional files.

## Additional files

Additional file 1
**Adjusted entry means for all assessed traits for eight inbred lines examined at three heat levels.**

Additional file 2
**MAPMAN bins of heat responsive genes.** MAPMAN bins of overall heat responsive genes, shown in figure 4, which were significantly (FDR <0.05 and |*log*_2_(*β*)|>2) differently expressed across all inbred lines with increasing heat levels, as well as the number of genes, that were allocated to each bin.

Additional file 3
**Description of heat responsive genes.** Overall heat responsive genes with MAPMAN bins shown in figure 4, which were significantly (FDR <0.05 and |*log*_2_(*β*)|>2) differently expressed across all inbred lines with increasing heat levels, with their bin description, log_2_ fold change (log _2_(*β*)), log_2_ counts per million, gene name, chromosome, as well as start and end position in the genome.

Additional file 4
**Significantly (FDR** ***<0.05*** **) enriched GO terms in the set of heat responsive genes.**

Additional file 5
**Correlation between RNA sequencing and qRT-PCR.** Log_2_ fold expression changes (FC) between 25°C and 32°C, 25°C and 38°C, as well as 32°C and 38°C of 11 genes determined by RNA sequencing and qRT-PCR.
