# Supplementary material for: Genome-wide expression profiling and phenotypic evaluation of European maize inbreds at seedling stage in response to heat stress
Source: BMC Genomics. 2015 Feb 25;16(1):123. doi: 10.1186/s12864-015-1282-1 (PMC4347969; doi:10.1186/s12864-015-1282-1)
Supplement: Additional file 1 — Adjusted entry means for all assessed traits for eight inbred lines examined at three heat levels. [file 12864_2015_1282_MOESM1_ESM.pdf]

Additional file 1 Adjusted entry means for all assessed traits for eight inbred lines examined at three heat levels

	Growth rate [cm/hour]			Dry weight [g]			Plant height [cm]			Number of leaves			Leaf temperature [°C]			Leaf greenness [SPAD]		
Inbred	25°C	32°C	38°C	25°C	32°C	38°C	25°C	32°C	38°C	25°C	32°C	38°C	25°C	32°C	38°C	25°C	32°C	38°C
L043	0.24	0.31	0.20	2.27	1.59	1.08	21.00	21.02	15.03	3.60	4.11	4.57	24.50	31.86	37.06	47.65	40.37	32.85
S058	0.21	0.23	0.28	1.54	0.85	0.51	17.75	15.90	10.30	3.90	4.90	4.80	24.47	31.25	35.93	43.25	38.47	28.97
L017	0.26	0.34	0.20	2.47	2.05	0.88	23.70	23.95	16.45	3.30	3.80	4.50	24.91	32.15	36.74	47.38	34.93	30.50
L023	0.26	0.30	0.23	2.26	1.65	0.78	20.80	18.90	13.22	3.30	4.20	4.90	24.98	31.95	36.89	48.66	37.97	32.52
L012	0.25	0.31	0.20	1.35	1.51	0.35	20.60	19.55	10.60	3.00	4.00	3.70	24.91	32.19	36.46	53.39	36.47	22.76
S067	0.20	0.33	0.16	1.62	2.17	0.68	23.17	24.90	14.85	3.99	4.70	5.58	24.52	31.80	36.57	43.19	21.97	28.59
S070	0.23	0.22	0.07	2.07	1.28	0.28	24.05	20.47	10.17	4.00	5.00	4.45	24.66	31.58	35.85	45.34	33.45	20.11
P040	0.28	0.27	0.14	2.57	1.33	0.38	23.70	20.03	9.50	3.20	4.67	4.02	24.90	31.91	35.28	47.53	30.33	39.00
