# Supplementary material for: Genome-wide expression profiling and phenotypic evaluation of European maize inbreds at seedling stage in response to heat stress
Source: BMC Genomics. 2015 Feb 25;16(1):123. doi: 10.1186/s12864-015-1282-1 (PMC4347969; doi:10.1186/s12864-015-1282-1)
Supplement: Additional file 4 — Significantly (FDR <0.05 ) enriched GO terms in the set of heat responsive genes. [file 12864_2015_1282_MOESM4_ESM.pdf]

Additional file 4 Significantly (FDR< 0.05) enriched GO terms in the set of heat responsive genes

**GO term**	Category	GO description
Upregulated		
GO:0009611	Biological process	response to wounding
GO:0004867	Molecular function	serine-type endopeptidase inhibitor activity
GO:0009605	Biological process	response to external stimulus
GO:0004866	Molecular function	endopeptidase inhibitor activity
GO:0030414	Molecular function	peptidase inhibitor activity
GO:0048046	Cellular component	apoplast
GO:0006073	Biological process	cellular glucan metabolic process
GO:0044042	Biological process	glucan metabolic process
GO:0044264	Biological process	cellular polysaccharide metabolic process
GO:0005976	Biological process	polysaccharide metabolic process
GO:0004553	Molecular function	hydrolase activity, hydrolyzing O-glycosyl compounds
GO:0034641	Biological process	cellular nitrogen compound metabolic process
GO:0016798	Molecular function	hydrolase activity, acting on glycosyl bonds
GO:0004857	Molecular function	enzyme inhibitor activity
GO:0006457	Biological process	protein folding
GO:0044262	Biological process	cellular carbohydrate metabolic process
GO:0005576	Cellular component	extracellular region
GO:0044106	Biological process	cellular amine metabolic process
GO:0016757	Molecular function	transferase activity, transferring glycosyl groups
GO:0005975	Biological process	carbohydrate metabolic process
GO:0006520	Biological process	cellular amino acid metabolic process
GO:0006519	Biological process	cellular amino acid and derivative metabolic process
GO:0009308	Biological process	amine metabolic process
GO:0016787	Molecular function	hydrolase activity
GO:0003824	Molecular function	catalytic activity
GO:0044238	Biological process	primary metabolic process
Downregulated		
GO:0042221	Biological process	response to chemical stimulus
GO:0016829	Molecular function	lyase activity
GO:0005506	Molecular function	iron ion binding
GO:0020037	Molecular function	heme binding
GO:0046906	Molecular function	tetrapyrrole binding
GO:0004175	Molecular function	endopeptidase activity
GO:0004713	Molecular function	protein tyrosine kinase activity
GO:0016491	Molecular function	oxidoreductase activity
GO:0003824	Molecular function	catalytic activity
