# Supplementary figures and images for: Genome-wide expression profiling and phenotypic evaluation of European maize inbreds at seedling stage in response to heat stress
Source: BMC Genomics. 2015 Feb 25;16(1):123. doi: 10.1186/s12864-015-1282-1 (PMC4347969; doi:10.1186/s12864-015-1282-1)

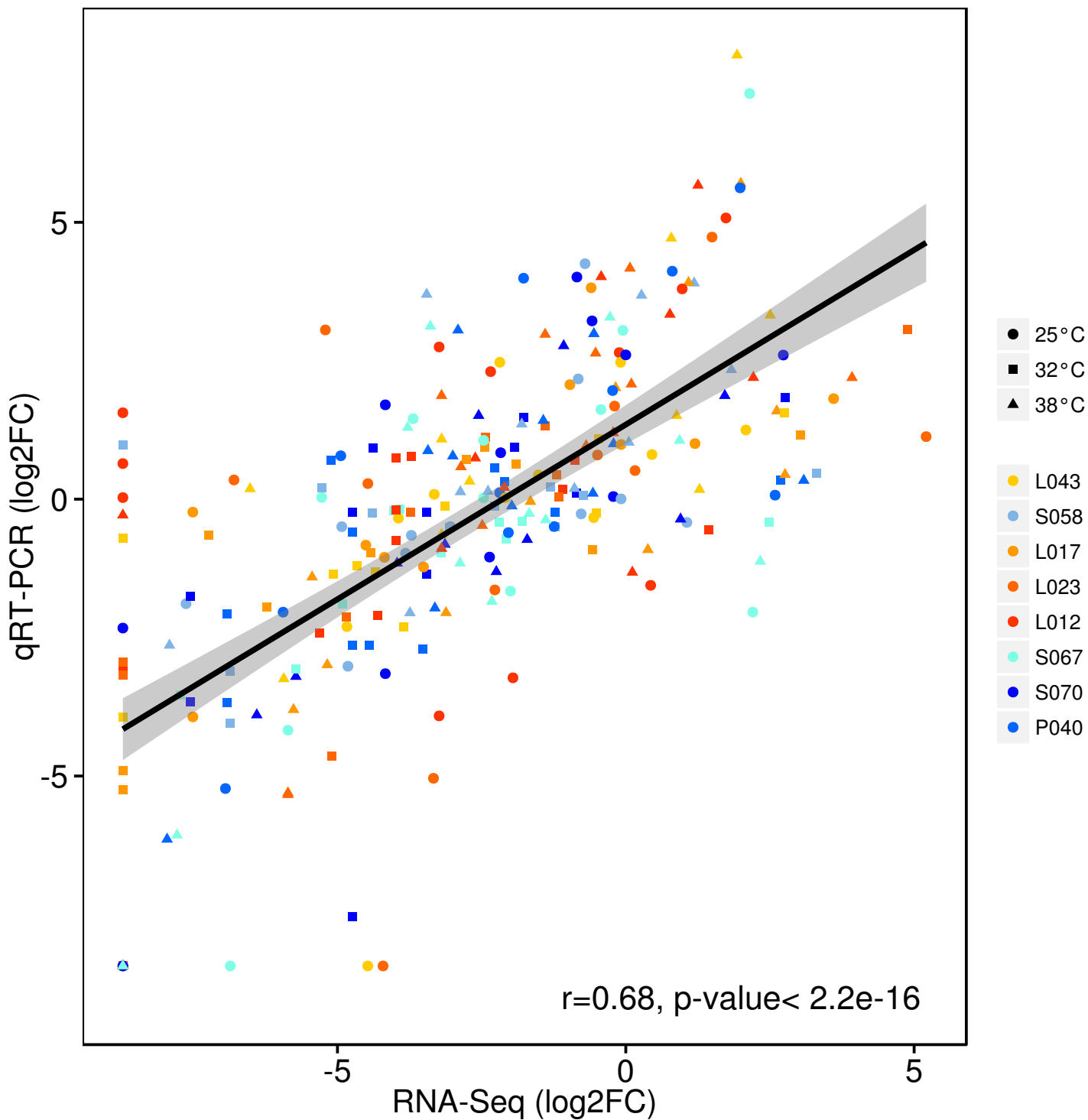

Supplement: Additional file 5 — Correlation between RNA sequencing and qRT-PCR. Log2 fold expression changes (FC) between 25°C and 32°C, 25°C and 38°C, as well as 32°C and 38°C of 11 genes determined by RNA sequencing and qRT-PCR. [file 12864_2015_1282_MOESM5_ESM.pdf]
